# Infliximab Biosimilar Utilization in a Large Pediatric Learning Health System

**DOI:** 10.3390/children12050656

**Published:** 2025-05-20

**Authors:** Ross M. Maltz, Shehzad A. Saeed, Jeremy Adler

**Affiliations:** 1 Department of Pediatrics, The Ohio State University Wexner Medical Center, Columbus, OH 43210, USA; 2 Division of Pediatric Gastroenterology, Hepatology, and Nutrition, Nationwide Children’s Hospital, Columbus, OH 43205, USA; 3 Department of Pediatrics, Wright State University Boonshoft School of Medicine, Dayton, OH 45404, USA; 4 Division of Pediatric Gastroenterology, Michigan Medicine, Ann Arbor, MI 48109, USA; jeradler@med.umich.edu; 5 Susan B. Meister Child Health Evaluation and Research Center, University of Michigan, Ann Arbor, MI 48109, USA

**Keywords:** ImproveCareNow, anti-TNF, inflammatory bowel disease

## Abstract

**Background/Objectives**: Infliximab biosimilars entered the United States (US) market in November 2016. Uptake of infliximab biosimilars has been slow in adult studies. We aimed to assess variation in the initiation of infliximab biosimilars in a large pediatric cohort. **Methods**: We performed a retrospective cohort study using data from 2016 to 2023 prospectively collected by the ImproveCareNow (ICN) Network, a multicenter pediatric inflammatory bowel disease (IBD) quality improvement collaborative. Pediatric patients with IBD who started any infliximab therapy were included. Descriptive statistics were used to summarize patient characteristics and changes in the use of infliximab agents. Chi-square or Fisher’s exact tests were used to evaluate differences in infliximab biosimilar initiation over time by race, age, ethnicity, and region. **Results**: In total, 4602 patients from 73 ICN centers started an infliximab agent. Infliximab biosimilar initiation rose steadily from 1% in 2018 to nearly 42% in 2023, with 88% of centers using biosimilars in 2023. Overall, from 2016 to 2023, the total percentage of patients who were started on an infliximab biosimilar was 17.3%. There were no differences in infliximab biosimilar initiation by age, race, or ethnicity, except in 2020 for age and race. The Midwest, West, and Southwest regions had higher initiation rates of infliximab biosimilars than the rest of the US. **Conclusions**: The percentage of patients with IBD initiating an infliximab biosimilar rose slowly to nearly 42% by 2023, and eight (12%) centers never recorded prescribing an infliximab biosimilar in the ICN Network. There were no differences in biosimilar initiation based on race or ethnicity.

## 1. Introduction

Tumor necrosis factor alpha inhibitors (anti-TNFs) have become first-line and second-line therapies for the treatment of patients with pediatric Crohn’s disease (CD) [1] and ulcerative colitis (UC) [2]. Access to United States (US) Food and Drug Administration (FDA) approved treatments for pediatric inflammatory bowel disease (IBD) continues to be substantially limited because while there are multiple medications with different mechanisms of action approved for adults with IBD, anti-TNFs are currently the only class of medications that are FDA approved for the treatment of children with IBD. The limited availability of FDA-approved medications and limited pediatric evidence base engenders reluctance to change prescribing patterns among the pediatric gastroenterology community.

Biosimilars are biologic products that are similar to the originator and have demonstrated similar efficacy and safety to the infliximab originator in adults and children with IBD [3,4,5,6,7,8,9]. The introduction of biosimilars was expected to decrease drug costs and improve treatment access [10,11]. The utilization had been slow due to limited data on the safety and efficacy of biosimilar switching, the need for education of physicians and patients, a change in provider prescribing habits, and regulatory hurdles [11,12,13]. Based on a Crohn’s and Colitis Foundation survey in 2018, pediatric IBD providers were less comfortable prescribing biosimilars compared to adult IBD providers [14]. This may be attributed to the relative novelty of biosimilar utilization in the pediatric IBD population, informed by the paucity of pediatric efficacy and safety studies.

This study assessed the initiation of infliximab biosimilars in a large pediatric cohort and evaluated whether there were differences in use by race, age, or region.

## 2. Materials and Methods

We performed a retrospective cohort study evaluating prospectively recorded registry data from the ImproveCareNow Network (ICN; www.ImproveCareNow.org) from children and young adults with IBD from 2016 through 2023. ICN is a learning health system, which is an international multicenter pediatric IBD quality improvement collaborative involving over 100 pediatric gastroenterology centers [15]. Individual ICN centers obtain consent from parents or patients (and, as appropriate, assent from patients) for use of data for research. Only data from consenting patients were included in this study. Clinicians at ICN centers prospectively collect data at all outpatient visits, including patient characteristics, laboratory values, and medications, which are entered into the central registry. This study was approved by ICN and the Institutional Review Boards at Nationwide Children’s Hospital in Columbus, OH (STUDY0002482) and C.S. Mott Children’s Hospital in Ann Arbor, MI (HUM00046270).

Only patients who were started on either the infliximab originator or an infliximab biosimilar (infliximab-dyyb, infliximab-abda, or infliximab-axxq) from 1 January 2016 to 31 December 2023 were included. The ICN registry does not have a variable for infliximab-axxq, but some centers did note in the medications when they were started on infliximab-axxq. As this study did not evaluate biosimilar switching, we reviewed patient data in 2015 to ensure we only included patients who had newly started infliximab and excluded all patients who had received infliximab prior to 2016. All patients were excluded from the six international sites within ICN due to the differential introduction of biosimilars. Patients were excluded from centers that had fewer than 10 patients who initiated infliximab throughout. Patients were excluded if the date of diagnosis, enrollment diagnosis, or date of birth were missing. Patients were also excluded if the enrollment date was before the date of diagnosis and if there was no recorded follow-up after initiating infliximab.

Patient demographics, including age at IBD diagnosis, age at infliximab initiation, IBD type and phenotype at infliximab initiation, sex, race, and ethnicity, were recorded. Descriptive statistics were used to summarize patient characteristics. Changes in the initiation of infliximab originator and each infliximab biosimilar were assessed across ICN sites from 2016 to 2023. The percentage of infliximab biosimilar initiation was evaluated by network, center, and by year.

Chi-square, Fisher’s exact test, or Wilcoxon rank sum test were used to evaluate differences in infliximab biosimilar initiation by age, IBD phenotype, race, ethnicity, region, extra-intestinal symptoms, and year. Race was categorized as White, Black, or other. Patients with unknown race were excluded from analyses of race. Age at diagnosis was categorized into three groups, including very early onset IBD (VEOIBD; <6 years old), pediatric (6 to <18 years old), and adult (≥18 years old). US regions were divided into Northeast, Southeast, Midwest, West, and Southwest.

## 3. Results

**Figure 1:**
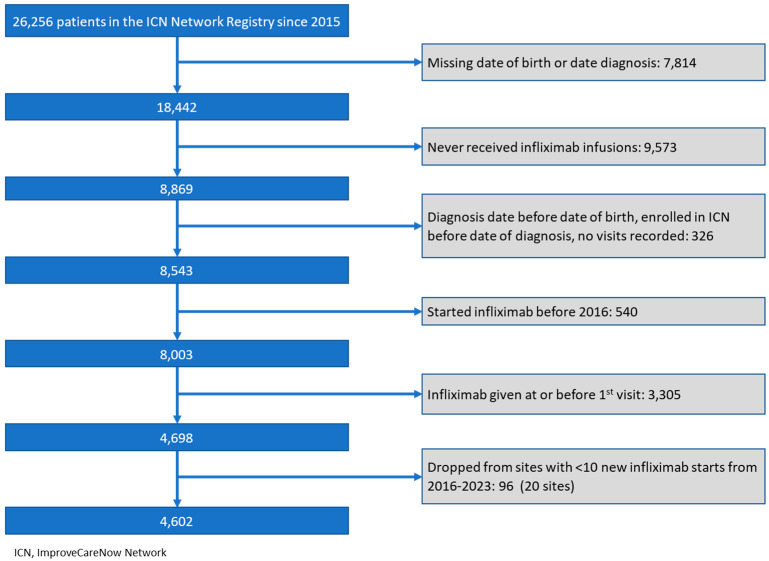
Inclusion and exclusion criteria.

At the time of the study, the ICN registry included 26,256 consenting patients with IBD from 100 participating centers. Ninety-nine centers were located in the US, and one center was located outside the US. Eighty-two percent (21,654) of patients were excluded because they did not meet the inclusion/exclusion criteria (Figure 1). The study population included a total of 4602 patients from 73 different ICN centers who started on an infliximab agent (Table 1). Forty-five percent were female, and the median age of infliximab initiation was 13.7 years (interquartile range [IQR] 8.3–16.2). Nearly 70% of patients had CD and 25% had UC. Over the study period, 3807 patients were started on infliximab originator and 795 patients were started on an infliximab biosimilar. There was no clinical significance between the two populations.

**Figure 2:**
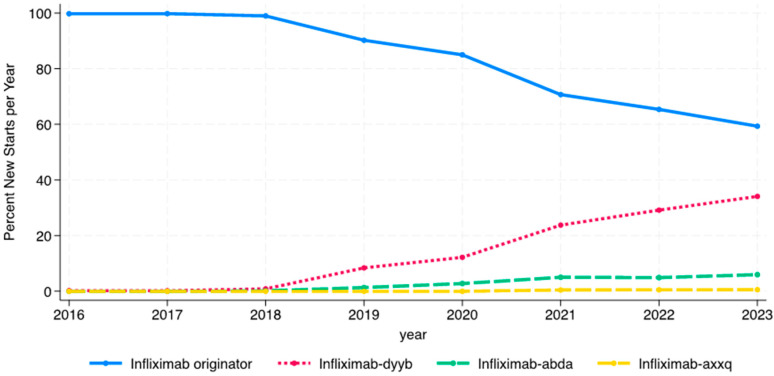
Variation in initiation of infliximab originator and biosimilars over time.

**Figure 3:**
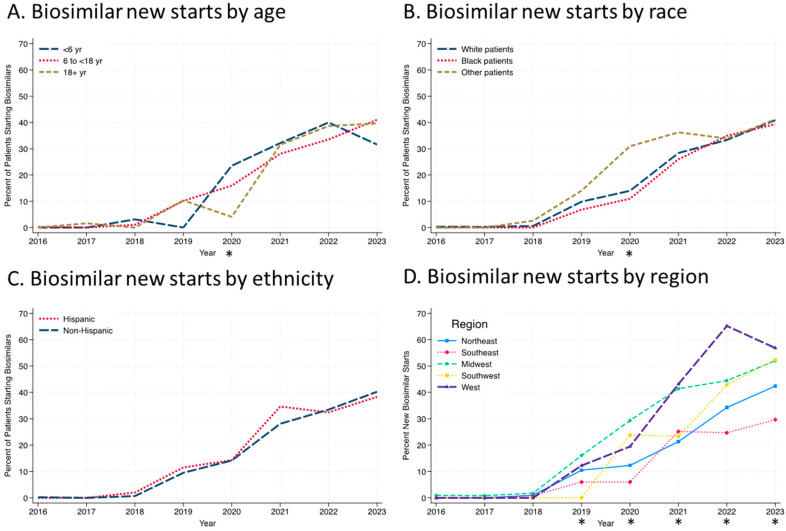
Infliximab biosimilar initiation by demographics: (**A**) by age, (**B**) by race, (**C**) by ethnicity, (**D**) by geographic region. * = *p* < 0.05.

While infliximab biosimilars entered the market in November 2016, no patients were started on a biosimilar in 2016, and only one patient was started on a biosimilar (infliximab-dyyb) in 2016 and 2017. The percentage of patients starting on infliximab biosimilars steadily increased from 1% of infliximab initiators in 2018 to nearly 42% in 2023 (Figure 2). The use of infliximab-dyyb outpaced the use of the other biosimilars. There were similar rates of infliximab biosimilar initiation by age group every year except in 2020, when there was a significant difference between the three groups (Figure 3A; *p* = 0.03). This is most likely due to the small number of patients initiating anti-TNFs in the <6 age group (n = 17) in 2020 and four patients starting on a biosimilar. Similarly, only in 2020 was there a significant difference by race, with patients grouped as “other” having a higher percentage of biosimilar use than Black or White patients (Figure 3B; *p* = 0.015). Throughout the study period, there were no differences in rates of biosimilar use by ethnicity (Figure 3C). Regional differences in infliximab biosimilar use were found (Figure 3D). In the Midwest, West, and Southwest regions, infliximab biosimilars were used at higher rates in 2020–2023 than in the Northeast and Southeast US (*p* < 0.05).

**Figure 4:**
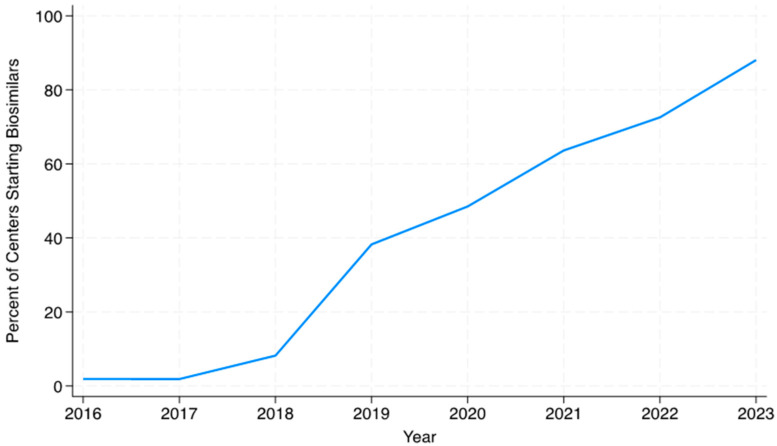
Infliximab biosimilar initiation by center over time.

Overall, from 2016 to 2023, only 17.3% of patients who initiated infliximab were started on an infliximab biosimilar. There was a slow uptake of infliximab biosimilar use among centers. Initially, only one center (1.9%) within ICN used an infliximab biosimilar in 2016, and five (8.2%) centers used an infliximab biosimilar in 2018. From 2019 to 2023, the percentage of centers using biosimilars increased from 38.2% (N = 26) to 88.1% (N = 59; Figure 4). Only three centers (4.1%) prescribed infliximab originator but never recorded prescribing any infliximab biosimilars to a patient throughout the 8 years of the study. The number of centers that had fewer than 10% of patients starting on an infliximab biosimilar in a year continued to decrease throughout the study. By 2023, nine centers (13.4%) had fewer than 10% of patients initiating infliximab, who started on an infliximab biosimilar. The number of centers that had between 25–69% and 70–100% of patients starting on an infliximab biosimilar per year increased from 11 (16.2%) and 3 (4.4%) centers, respectively, in 2019 to 35 (52.2%) and 19 (28.4%) centers in 2023.

## 4. Discussion

Infliximab biosimilar initiation in a large pediatric network was slow at first, and increased to nearly 41% of the infliximab new starts by 2023. To the best of our knowledge, this is the first large multicenter cohort study to evaluate infliximab biosimilar initiation in pediatric patients with IBD. Infliximab biosimilar utilization has been slow in the US. Hussaini et al. showed that the utilization in adults on Medicare Part B only increased to 3.6% by 2019, which is less than what we found (10.8%) in the pediatric ICN Network [16]. In a recent study utilizing Merative MarketScan Commercial claims, we found that biosimilar utilization had only 9% of the market in 2019 and increased to 26% by the end of 2021 for new infliximab starts, which is similar to this study (manuscript in press). This data captured 42,000 pediatric and adult patients up to 64 years old starting on infliximab regardless of the diagnosis.

Some centers were early adopters of biosimilars, and the number of centers that started to use biosimilars continued to increase. The exact reasons why a large percentage of centers within the ICN Network were initially slow to use infliximab biosimilars are unknown. Maltz et al. similarly found in a 2021 survey that among pediatric gastroenterologists, 21% were not prescribing anti-TNF biosimilars. The most common reasons reported by respondents were that they preferred the originator drug, were unsure of the efficacy of biosimilars, were uncomfortable prescribing biosimilars, or were not aware if biosimilars were on their institutional formulary [13]. Respondents reported that they felt additional education and pediatric-specific data would make them more comfortable prescribing biosimilars. The survey also found that pediatric gastroenterologists from ICN centers were more comfortable prescribing anti-TNF biosimilars compared to pediatric gastroenterologists who were not practicing at ICN participating centers. Despite concerns amongst some pediatric gastroenterologists, infliximab biosimilars are proven to have similar efficacy and safety to the originator in adults with IBD [3,7,17,18,19]. While studies in pediatric patients with IBD are limited, they have also largely found similar efficacy and safety of the infliximab biosimilar to the originator among patients newly initiating infliximab [4,5,6,8,9]. Avoiding infliximab biosimilar use is associated with increased cost of care since infliximab biosimilars were initially priced 10–50% less than the originator drug [4,20,21,22].

Unfortunately, we were not able to assess the reasons for the slow initiation of infliximab biosimilars across the ICN Network. Overall, the patients that started on infliximab originator or a biosimilar were clinically similar. Only in 2020 did we find a difference in biosimilar initiation based on race or ethnicity. It is possible that the COVID-19 pandemic affected this. We were unable to evaluate whether there were disparities based on race or ethnicity within individual centers due to the small sample size. Disparities, if present, may also be related to socioeconomic status. Unfortunately, assessing socioeconomic status and health insurance type was beyond the scope of this study due to a lack of such data in the ICN registry. In 2020, there was also a statistically significant difference in biosimilar initiation based on age. Patients who were less than 6 years old had the highest percentage of biosimilar initiation, and patients who were older than 18 years old had the lowest percentage. However, there were only 17 patients under 6 years old that year, so this finding was most likely driven by the small sample size. By 2021, there were no differences in the percentage of biosimilar initiation between the three age groups. Patients with VEOIBD commonly have rapid drug clearance and may require higher doses to obtain therapeutic levels than older children [23]. There were regional differences in biosimilar initiation in 2020–2023, with the Midwest, West, and Southwest US regions having the highest percentages of biosimilar initiation compared to the Northeast and Southeast regions. The reason for this remains to be explored. While this is beyond the scope of our study, we suspect institutional acceptance of biosimilars and/or individual state insurance policies affected regional differences.

There were a few limitations in our study worth noting. The recording of clinical data in the ICN Network registry is dependent on local processes to consent individuals to use data for research and accurately capture the type of infliximab product used. This could create a degree of bias because larger centers and centers with more resources may be more likely to be able to consent more patients for research as compared to smaller centers and centers with resource constraints. Due to the nature of the point of care and real-world data capture, we are unable to verify the accuracy of the data. If centers did not differentiate infliximab products, it could potentially lead to the undercounting of biosimilar use, especially infliximab-axxq, which is not a variable in the network unless centers commented on their use. However, the proportion of biosimilar use is similar to that of national administrative claims data (manuscript in press). We assessed disparities by race and ethnicity, but 8% of patients were not included in the analyses due to missing race, and 4.6% due to missing ethnicity, which may introduce bias. Individual centers each establish their own processes for entering data into the network, and we cannot determine the reliability of race and ethnicity data recorded in the ICN registry [24]. We wanted to evaluate whether the insurance type affected biosimilar initiation, but unfortunately, the insurance type was entered into the registry at diagnosis, and there was no record of insurance at the time of infliximab initiation. Thus, patients could change insurance type over time, and the data captured in the ICN registry regarding insurance type may not reflect the current state. A notable strength is that data in the ICN Network registry are collected prospectively by clinicians. The ICN Network represents one of the largest pediatric IBD registries in existence and includes both large academic medical centers as well as small hospital-based and private practices, truly reflective of the real-world nature of pediatric IBD care.

## 5. Conclusions

Infliximab biosimilar initiation has increased, but there were variations in biosimilar initiation amongst centers in the ICN Network. Multiple centers were initially slow at utilizing infliximab biosimilars. There were regional differences, but factors such as age, race, and ethnicity had little impact on biosimilar initiation. Further research is needed about the clinical impact, treatment longevity, and changes in cost associated with large-scale biosimilar use in pediatric IBD.

## Figures and Tables

**Table 1:** Baseline demographics.

	All Patients	Infliximab Originator	Infliximab Biosimilar	*p*-Value
	N = 4602	N = 3807	N = 795	
Median age at IBD diagnosis	12.6 (IQR 9.8–15.2)	12.6 (IQR 9.7–15.3)	12.8 (IQR 10.2–15.0)	0.48
Median age at start of infliximab	13.7 (IQR 10.9–16.2)	13.6 (IQR 10.8–16.3)	13.8 (IQR 11.4–16.0)	0.46
Female	2085 (45.3%)	1742 (45.8%)	343 (43.1%)	0.18
**Ethnicity, n (%)**				
Hispanic	298 (6.5%)	241 (6.3%)	57 (7.2%)	0.13
Non-Hispanic	4093 (88.9%)	3401 (89.3%)	692 (87.0%)	
Missing	211 (4.6%)	165 (4.3%)	46 (5.8%)	
**Race, n (%)**				
Black/African American	718 (15.4%)	594 (15.6%)	124 (15.6%)	1.00
White	3359 (71.9%)	2798 (73.5%)	561 (70.6%)	0.091
Asian	149 (3.2%)	111 (2.9%)	38 (4.8%)	0.007
Other/Multiracial	52 (1.1%)	1.2% *	1.0% *	0.72
Unknown	389 (8.3%)	311 (8.2%)	78 (9.8%)	0.13
**Diagnosis, n (%)**				
Crohn’s disease	3145 (68.3%)	2615 (68.7%)	530 (66.7%)	0.001
Ulcerative colitis	1171 (25.5%)	938 (24.6%)	234 (29.4%)	
IBD-unclassified	285 (6.2%)	254 (6.7%)	31 (3.9%)	
**Crohn’s Disease location, n (%)**				
L4a	1425 (45.3%)	1212 (46.4%)	213 (40.2%)	<0.001
L4b	754 (24.0%)	648 (24.8%)	106 (20.0%)	<0.001
L1	492 (15.7%)	410 (15.7%)	82 (15.5%)	0.46
L2	580 (18.4%)	479 (18.3%)	101 (19.1%)	
L3	1929 (61.3%)	1611 (61.6%)	318 (60.0%)	
Missing	78 (2.5%)	59 (2.3%)	19 (3.6%)	
None	66 (2.1%)	56 (2.1%)	10 (1.9%)	
**Crohn’s Disease phenotype, n (%)**				
B1	2441 (77.6%)	2048 (78.3%)	393 (74.2%)	0.082
B2	234 (7.4%)	196 (7.5%)	38 (7.2%)	
B3	323 (10.3%)	259 (9.9%)	64 (12.1%)	
B2/B3	35 (1.1%)	1.0% *	1.7% *	
Unknown	112 (3.6%)	86 (3.3%)	26 (4.9%)	
**Perianal phenotype, n (%)**				
Yes	845 (26.9%)	702 (26.9%)	143 (17.0%)	0.30
No	2223 (70.7%)	1854 (70.9%)	369 (69.6%)	
Missing	77 (2.5%)	59 (2.3%)	18 (3.4%)	
**Extent of disease-ulcerative colitis, n (%)**				
E1	52 (4.4%)	5.0% *	2.1% *	0.002
E2	159 (13.6%)	124 (13.2%)	35 (15.0%)	
E3	94 (8.0%)	72 (7.7%)	22 (9.4%)	
E4	832 (71.0%)	675 (72.0%)	157 (67.1%)	
Missing	35 (3.0%)	20 (2.1%)	15 (6.4%)	
Severe Behavior, n (%)	313 (26.7%)	247 (26.3%)	66 (28.2%)	0.39
**Extra-intestinal**				
Arthritis	214 (4.7%)	184 (4.8%)	30 (3.8%)	0.20
Growth Status in failure	123 (2.7%)	174 (4.6%)	19 (2.4%)	0.38
Pyoderma gangrenosum	20 (0.4%)	0.5% *	0.3% *	0.39
Uveitis	32 (0.7%)	0.7% *	0.4% *	0.26
Erythema Nodosum	39 (0.8%)	29 (0.8%)	10 (1.3%)	0.17

IBD, inflammatory bowel disease; L1, ileal only; L2, colonic; L3, ileocolonic; L4, upper disease; B1, inflammatory; B2, stricturing; B3, internal penetrating; B2/B3, stricturing and internal penetrating; IQR, interquartile range; E1 ulcerative proctitis; E2 left sided colitis (distal to splenic flexure); E3 Extensive colitis (hepatic flexure distally); E4, pancolitis (proximal to hepatic flexure). * Number of patients less than 10 are not included to protect patient confidentiality.

## Data Availability

Data can be made available by contacting the corresponding author.

## Abbreviations

The following abbreviations are used in this manuscript:

Anti-TNFs	Tumor necrosis factor alpha inhibitors
CD	Crohn’s disease
UC	Ulcerative colitis
US	United States
FDA	Food and Drug Administration
IBD	Inflammatory bowel disease
ICN	ImproveCareNow
IQR	Interquartile range
